# INITIAL BREASTFEEDING DIFFICULTIES AND ASSOCIATION WITH BREAST DISORDERS AMONG POSTPARTUM WOMEN

**DOI:** 10.1590/1984-0462/;2017;35;3;00004

**Published:** 2017-07-13

**Authors:** Gessandro Elpídio Fernandes Barbosa, Victor Bruno da Silva, Janeide Mendes Pereira, Marianne Silva Soares, Rosemberg dos Anjos Medeiros, Luciana Barbosa Pereira, Lucinéia de Pinho, Antônio Prates Caldeira

**Affiliations:** a Universidade Estadual de Montes Claros (Unimontes), Montes Claros, MG, Brasil.

**Keywords:** Breastfeeding, Lactation disorders, Risk factors

## Abstract

**Objective:**

To investigate the prevalence of difficulties in adopting initial breastfeeding techniques and their association with breast disorders in postpartum women.

**Methods:**

The cross-sectional study was carried out with 276 randomly selected mother-baby pairs in rooming-in in 3 hospitals in a city of Minas Gerais State (southeast Brazil). An assessment protocol was established to evaluate the breastfeeding technique used. The association between the variables studied and breast disorders was determined by the chi-square test followed by logistic regression, with significance level set at 0.05.

**Results:**

The main factors indicating difficulties to initiate the breastfeeding techniques were inadequate attachment of the baby to the breast (25%), baby response to the contact with the breast (26.1%) and breast disorders (28.3%). Variables associated with postparturm breast disorders were: adolescent mothers (OR 3.35; 95%CI 1.51-7.44; *p*=0.003); maternal schooling ≤8 years (OR 2.07; 95%CI 1.01-4.23; *p*=0.048); and supplement provision to the newborn at the hospital (OR 2.36; 95%CI 1.40-4.92; *p*=0.003). Mothers working outside the household (OR 0.31; 95%CI 0.16-0.61; *p*=0.001) served as as protective factor on the multivariate model.

**Conclusions:**

The main difficulties in initial breastfeeding were associated with breast disorders, and the factors associated with this problem included demographic and social, variables, as well as others related to the care routine adopted by maternity hospitals.

## RESUMO

**Objetivo:**

Identificar a prevalência de condições indicativas de dificuldades iniciais com a técnica da amamentação e verificar os fatores associados com a presença de problemas na mama em puérperaso em maternidades de Hospitais Amigos da Criança.

**Métodos:**

Estudo transversal, conduzido com 276 binômios mãe-lactente, aleatoriamente selecionados entre os atendimentos realizados em maternidades de três Hospitais Amigos da Criança do norte de Minas Gerais. Um protocolo de avaliação da técnica da mamada foi utilizado como instrumento. A associação entre as variáveis estudadas e os problemas com a mama foi identificada por meio do teste qui-quadrado, seguida de análise de regressão logística, admitindo-se o nível de significância de até 5% (*p*<0,05).

**Resultados:**

Os principais fatores indicativos de dificuldades iniciais com a técnica da amamentação foram a pega inadequada (25,0%), a resposta do bebê ao contato com a mama (26,1%) e os problemas com a mama (28,3%). No modelo final de regressão múltipla, associaram-se aos problemas na mama no puerpério imediato: mãe adolescente (OR 3,35; IC95% 1,51-7,44; *p*=0,003); escolaridade ≤8 anos (OR 2,07; IC95% 1,01-4,23; *p*=0,048); e o fato de ter recebido complemento alimentar na maternidade (OR 2,36; IC95% 1,40-4,92; *p*=0,003). O fato de trabalhar fora de casa (OR 0,31; IC95% 0,16-0,61; *p*=0,001) foi um fator de proteção no modelo final de regressão logística.

**Conclusões:**

Problemas com as mamas representaram a principal dificuldade inicial com a técnica da mamada e os fatores associados incluem variáveis demográficas e sociais ligadas à mãe e variáveis relacionadas às rotinas da maternidade.

## INTRODUCTION

The World Health Organization (WHO) recommends exclusive breastfeeding (EBF) for six months, and complemented until the age of 2 or more, considering the benefits proved in practice for both mother and child.^1^ The Ministry of Health in Brazil has the same recommendation, and the country has one of the most advanced legislations for maternal breastfeeding in the world, ensuring several rights to women and providing favorable conditions for breastfeeding.^2^ Despite the recommendations and the measures adopted, early weaning, which is the interruption of breastfeeding before the newborn has completed 6 months of age, regardless of the reason, is still a frequent and undesirable reality.^3^ ^,^ ^4^

An early weaning facilitator is still little explored in national and international literature, and is related to the difficulties inherent to the breastfeeding technique. It is believed that a poor technique would make it difficult for the newborn to suck and empty the breast, thus affecting the dynamics of milk production. As a consequence, the mother can introduce other foods early, causing the weaning.^5^ ^,^ ^6^ ^,^ ^7^

There are some very relevant aspects in the process of sucking that should be carefully assessed by health professionals in the activities for education and promotion of breastfeeding. Some behaviors observed during breastfeeding in the maternity hospital are not desirable, and seen as risk factors for weaning.^8^ ^,^ ^9^ Presence of nipple pain, mammary ingurgitation, mammillary lesion, fatigue and tiredness are examples of conditions that indicate difficulties with the breastfeeding technique, commonly mentioned in the first 24 hours postpartum. Besides these, other circumstnaces also have a negative impact on the duration of maternal breastfeeding, such as the presence of difficulties in handle and suction, the baby’s agitation and the perception of insufficient milk by the mother.^9^ ^,^ ^10^ ^,^ ^11^

WHO, together with the United Nation’s Children Fund (Unicef), recommend the use of a “sucking evaluation file” as a strategy to monitor and identify these initial difficulties involving the breastfeeding technique.^12^ Despite being little used, this instrument allows assessing behaviors that are favorable or not in relation to breastfeeding, including the mother and the newborn’s posture, the responses of the pair at the beginning of sucking, the establishment of affectional bonds, the characteristics of suction, the anatomical conditions of the breast, duration and conclusion of the sucking.^12^ ^,^ ^13^ ^,^ ^14^ This study aimed at identifying the prevalence of conditions indicating initial difficulties with breastfeeding, based on the use of a sucking evaluation file, and the factors associated with the presence of breast problems among mothers in the puerperium, in maternity wards of Hospitais Amigos da Criança, in the North of Minas Gerais.

## METHOD

This is a cross-sectional, observational and analytical study, which assessed mother-baby pairs, selected randomly, in the first 18 to 48 hours postpartum. The study was conducted in three hospitals, all named “Hospital Amigo da Criança”, in the north of Minas Gerais. The city where the study was conducted has about 390 thousand residents, and is a macro-regional reference for several sectors of the regional economy, as well as in the health field. Sample selection was random, restricted to mother-baby pairs assisted by the Unified Health System (SUS), who stayed in a collective accommodation after postpartum care and presented with conditions for being discharged from the hospital together.

Sample calculation was conducted to define the minimum number of pairs analyzed in the study, considering a population of 3,000 mothers thorughout a six-month period, with prevalence of initial problems involving the breast of 20%,^13^ sampling error of 5% and 95% confidence intervals (95%CI). A 10% rate was added for possible losses. Therefore, the minimum number of pairs approached should be 251. The days for data collection in each hospital were defined randomly.

The following inclusion criteria were considered: mothers who received postpartum care in one of the three hospitals in the city, with term pregnancy, who were in good health conditions, according to clinical records, to respond to the initial survey. Regarding newborns, the inclusion criteria were: good health conditions, according to clinical records, with possibility for hospital discharge and being exclusively fed with breast milk at the moment of discharge (the possible consumption of supplements in the maternity hospital was not considered as an interruption of exclusive breastfeeding). Exclusion criteria were: mothers who have not received immediate postpartum care (home parturition), mothers pregnant with twins and those whose did not stay in the group accommodation. The pairs were assessed at a hospital environment (accommodation), when considered to have conditions for hospital discharge. The evaluation was conducted from 18 to 48 hours postpartum.

The allocation of pairs was conducted by random selection on days and shifts for visits in each hospital, including all mothers who met the inclusion criteria and who accepted to participate. The allocation of the binomials for the study was conducted according to the proportion of births in the six months prior to the beginning of data collection in each hospital. The interviews were conducted by a previously trained and calibrated team, using the consensus technique by an obstetric nurse with experience in maternal breastfeeding and who is a monitor of the training course in Hospital Amigo da Criança in Counseling for Maternal Breastfeeding.

The data collection instrument included the sucking evaluation file,^12^ which contemplates, among other elements, the evaluation of the breasts, filled out by the direct observation of breastfeeding. Other variables were also analyzed in data colletion: social and demographic data (age, self-reported race, schooling, paid activity, family income, marital status, number of residents in the household, and presence of maternity leave), data about the newborn (sex, weight at birth, Apgar score at 1 and 5 minutes), besides information regarding gestational aspects, prenatal care and partum and puerperium assistance (parity, type of partum, number of prenatal appointments, information about care with the brests, permanence in a group accommodation, among others).

The Statistical Package for the Social Sciences (SPSS), version 18 (SPSS Inc., Chicago, IL, USA) was used for data analysis. The variables were assessed in a descriptive manner, by presenting absolute and relative frequencies. For the analytical stage of the analysis, problems with the breasts (engorged and firm; flat or inverted nipples; breasts or nipples with excoriations, fissures or redness) were analyzed and defined as a single outcome variable (dependent). The associated variables were identified among the other characteristics that were investigated (independent), based on the chi-square test. The variables associated to a 20% level (*p*<0.2) were assessed by the binary logistic regression, using the Backward Wald method. In this last stage, the Odds Ratio (OR) were calculated with the respective 95%CI. For the final model, only the variables associated up to a 5% level (*p*<0,05) were considered.

The study was conducted according to the ethical precepts, with strict attention to Resolution 466/2012. The project was assessed and approved by the Research Ethics Committee at Universidade Estadual de Montes Claros, number 844,557, and all mothers that took part signed the informed consent form.

## RESULTS

Two-hundred and seventy-six mother-baby pairs or binomials were assessed. Most mothers were aged between 20 and 29 years old, and the percentage of teenage mothers was 11.6%. The prevalent self-reported skin color was brown. More than half of the mothers reported family income of up to 1 minimum wage. Regarding schooling, most mothers reported having concluded from 5 to 8 years of schooling. Other sociodemographic aspects are demonstrated in Table 1.

**Table 1:**
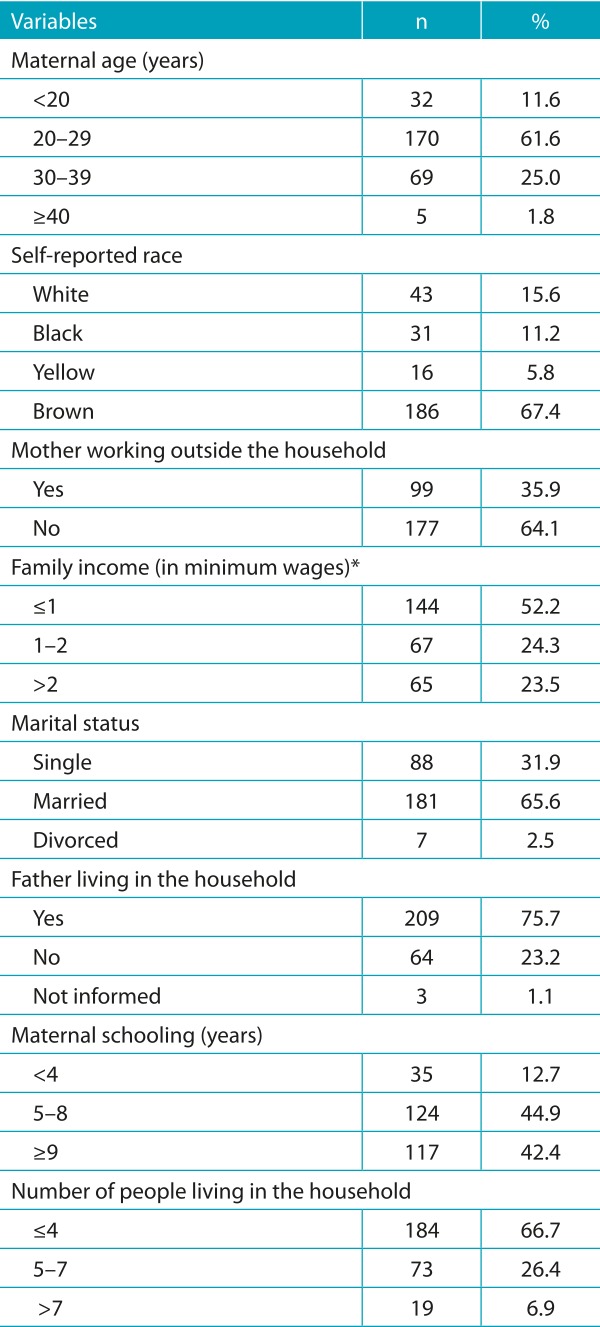
Demographic and socioeconomic characteristics of puerperal women; Montes Claros (MG), 2015.

*Current minimum wage = R$ 788.00 (around US 250.00).

About the features regarding the pregnancy and prenatal care (Table 2), most mothers were primigravid, and 55.1% of births were natural. The public health system was the most used one. 57.6 and 55.5% were advised as to breastfeeding and breast care during the prenatal period, respectively.

**Table 2:**
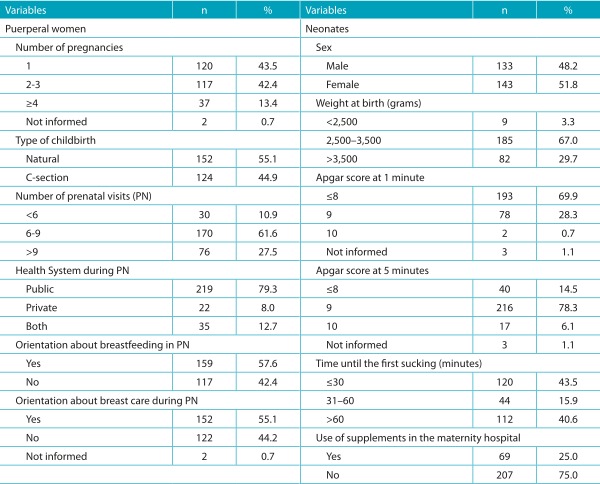
Characteristics of gestational and prenatal care of puerperal women and characteristics of the newborns; Montes Claros (MG), 2015.

Less than half of the newborns was breastfed in the first thirty minutes postpartum (43.5%), and the use of supplements for the child in the maternity hospital was reported by 25.0% of the mothers (Table 2)(t6).

The observation of the sucking allowed identifying conditions that indicated initial difficulties with the techniques in all the assessed aspects, especially inadequate handle (25.0%), response to the contact with the breast (26.1%), and problems with the breast (28.3%) (Table 3).

**Table 3:**
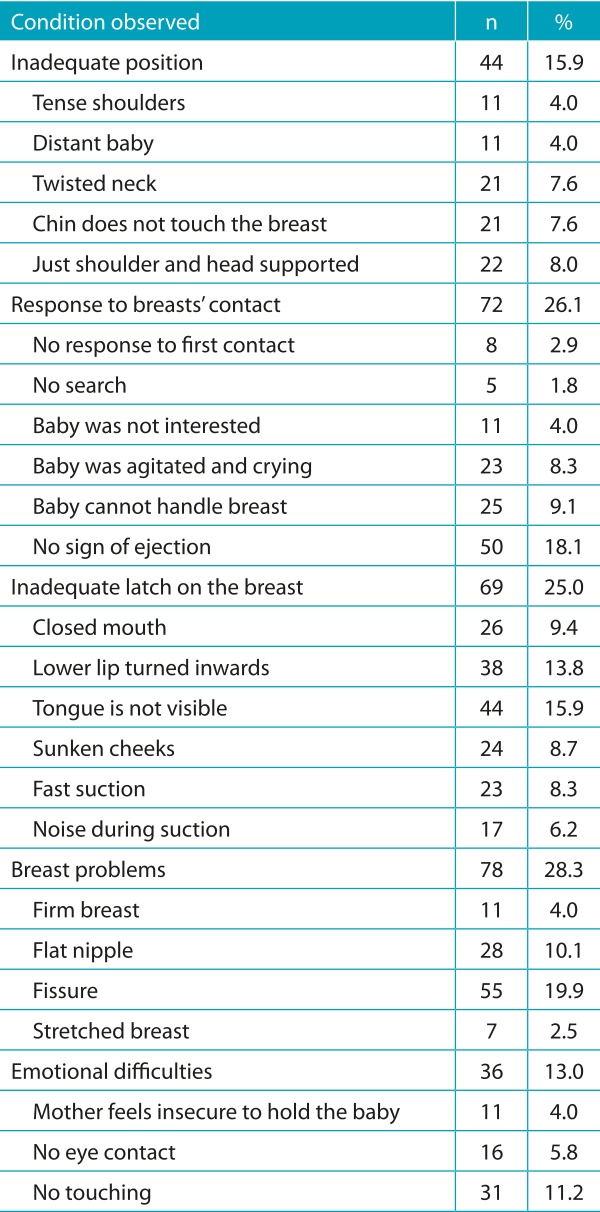
Prevalence of conditions indicating initial difficulties with the breastfeeding technique among puerperal women; Montes Claros (MG), 2015.

Table 3 registers the result of the bivariate analyses for the maternal and care characteristics, as well as the presence of breast problems. The variables in this table that were associated until up to 20% (*p*<0.2) were assessed together and remained in the final multivariate model as variable associated with breast problems: the fact of being a teenage mother (OR 3.35; 95%CI 1.51-7.44; *p*=0.003), schooling equal to or lower than 8 years (OR 2.07; 95%CI 1.01-4.23; *p*=0.048), the fact of having received supplements at the maternity hospital (OR 2.36; 95%CI 1.40-4.92; *p*=0.003) and the fact of working outside the household, which was a protective factor (OR 0.31; 95%CI 0.16-0.61; *p*=0.001) in the final logistic regression model.

**Table 4:**
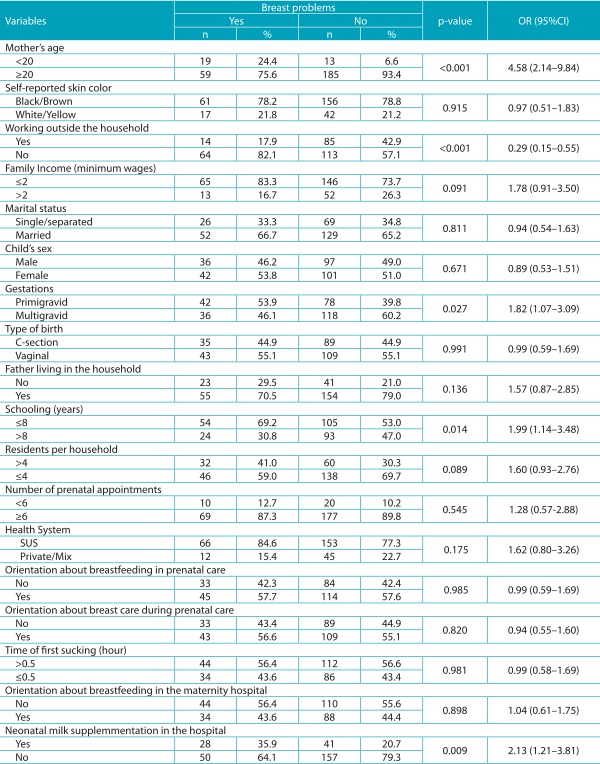
Association between difficulties in breastfeeding and the studied variables (bivariate analysis); Montes Claros (MG), 2015.

## DISCUSSION

The results obtained in the study show high prevalence of conditions indicating initial difficulties with the breastfeeding technique. Even though there are few studies described in the literature with the same approach, other authors also revealed high prevalence of initial breastfeeding difficulties by applying the sucking evaluation file from Unicef.^8^ ^,^ ^13^ ^,^ ^14^ ^,^ ^15^ This process may reflect the holistic aspect of the instrument used, since the sucking evaluation file includes different features, not only those related to sucking.

Carvalhaes et al., in a study conducted in a public maternity hospital working on low-risk childbirth, assessed 50 mother-baby binomials and showed that 18 to 34% of the pairs presented difficulties with the initial breastfeeding period, in at least one of the aspects of sucking that were observed.^13^ Pereira, in 2008, observed a 50% prevalence of breastfeeding difficulties in the maternity hospital, in a sample of 60 cases of mother-baby dyads.^15^ It is important to mention that even though the author also used the Unicef form, some mothers were advised by health professionals in relation to the breastfeeding technique when they were in the third trimester of pregnancy, and this previous training could also have influenced the numbers. Mosele et al. also used the evaluation protocol by Uniced and showed, based on the analysis of 152 mother-baby binomials admitted to a group accommodation, that 55% of the pairs presented at least one difficulty regarding breastfeeding. The main difficulties found were: “mother with tense sholders and leaning over the baby”, “baby cannot handle the nipple”, “mammary tissue with excoriations, mammillary lesion or redness” and “suction: mouth nearly closed, pointing outward, lower lip inside; you cannot see the baby’s tongue, with tense or sunken cheeks”.^14^

Traditionally, the most prevalent problems with puerperal breasts, that is, mammary ingurgitation and mammillary lesions, are attributed to the inadequate position for breastfeeding and/or the baby’s handle.^10^ In a case-control study conducted with women admitted to a university hospital in the state of São Paulo, involving binomials in which the children had twisted neck, chin away from the breast and lower lip turned inside, there was 1.9, 2.9 and 4.2 times more chances of presenting with mammillary trauma during breastfeeding, respectively, in comparison with binomials who did not have these characteristics.^8^ These results highlight the importance of using a sucking evaluation file to help identify problems with the breastfeeding technique, especially those related with inadequate handle, baby’s response to the contact with the breast and breast problems.

Breast problems may compromise the success of maternal breastfeeding. A national study identified a high incidence of mammillary lesions in the maternity hospital, of 43.6%.^7^ A prospective study carried out in Malasia showed breastfeeding difficulties owed to breast problems, such as lesion and mammillary pain, as major predictive factors for the interruption of exclusive maternal breastfeeding.^16^

In this study, the authors chose to assess, specifically, the factors associated with the presence of brast problems in mothers in the first 24 hours postpartum, still inside the maternity hospitals, because the changes could be identified more easily, with minor chances of subjective assessment by the evaluators. Therefore, the variables associated with breastfeeding difficulties were mother’s age and schooling, the fact that the newborn received supplements in a hospital environment and the report that the mother worked outside the household, being the later a protective factor. Literature did not show studies associating the mother’s age with breastfeeding problems. There are, however, records that teenage mothers provide less breast milk to their children in relation to the others, and also have more difficulties to initiate the practice of breastfeeding.^17^ ^,^ ^18^ ^,^ ^19^ However, the studies do not refer to breastfeeding aspects among the teenagers assessed. It is possible that anatomical factors of the puerperal breasts of teenagers may lead to more vulnerability regarding the difficulties presented, but this aspect was not assessed in this study. Considering that maternal age is a non-modifiable factor for breastfeeding, the result observed highlights that education regarding breast care should be reinforced among younger women, aiming at improving the breastfeeding practices.

It was not possible to identify scientific articles that associate the use of food supplement still in the hospital with breast problems, even though an association has been observed with worse scores in the breastfeeding file.^13^ Chantry et al. demonstrated that the use of food supplements in the hospital was related to the presence of pain while breastfeeding.^20^ It is not possible to establish a cause-effect relation between both events, and the association observed could be a result of the reverse causation effect, that is, the fact of presenting breast problems would be the trigger for the use of food supplements still in the maternity hospital. Anyway, the intake of any other food during the first months of breastfeeding, besides being a major risk factor for the development of illnesses that are common in childhood, such as pneumonia or diarrhea, also increases the chances of early weaning, since it interferes in some aspects of the sucking technique.^21^ ^,^ ^22^ This fact brings out the need for more rigor in hospital institutions while offering food supplements for newborns.

As to the association between breast problems and maternal schooling, other studies have indicated an intrinsic relationship between the practice of breastfeeding and schooling.^19^ ^,^ ^23^ Women with higher schooling tend to be more motivated to breastfeed for a longer period, maybe for having more access to information about the benefits and advantages caused by maternal breastfeeding for the binomial.^19^ It is likely that women with higher schooling also have more motivation to care for their breasts during gestation; therefore, women with lower schooling would present more breast problems in the initial stages of breastfeeding.

This study showed that the maternal report of working outside the household functions as a protective factor for the occurrence of problems with the puerperal breast. Even though we did not find other studies approaching this subject, the circumstance observed may be explained by the fact that working mothers have more opportunities to access positive information about breast care outside the household.

According to Roig et al., the information related to breast care and breastfeeding provided by health professionals during prenatal care are closely related with the success in maternal breastfeeding, as well as with the presence of positive previous experiences with breastfeeding.^24^ Therefore, this study did not show a significant association between the fact that the mother received orientation during prenatal care regarding breastfeeding or breast problems, and the prevalence of initial problems with the breast in the first 18 to 48 hours postpartum. This fact can be justified by the lack of parameters that could characterize an adequate offer - or not - of information and the communication between the prenatal staff and the pregnant woman. The former may have given the orientation, but without significant emphasis during the appointments; there might have been a limitation in the pregnant woman’s memory about the orientation.

The results of this study including some limitations, such as the fact that information regarding previous lactation experiences were not considered. This fact could be related to the lower prevalence of initial difficulties among multigravid women, but this data was not measured alone. Another limitation is that only women assisted in the Unified Health System were assessed, even if many of them have received prenatal care in the private system, or both. Therefore, the prevalence of initial difficulties may have been measured only in the lower social strata of the population, and the results of this study, therefore, may not be extrapolated for binomials assisted by the private health system.

The evaluation of difficulties in sucking techniques, still in the hospital environment, is a simple, economic measure that does not require a specialized professional. Therefore, it can be incorporated to the criteria of hospital discharge, in order to identify and assist the binomials that present some sort of problem regarding the breastfeeding process, providing the adequate orientation to solve these difficulties and to strengthen the mother-baby connection.

As a future perspective, the determination of the impact of initial difficulties with the sucking technique on the duration of exclusive maternal breastfeeding should be assessed by the longitudinal observation of the mother-baby pair, and that may show exactly which are the factors related with the sucking technique that can be related with early weaning.
